# Burden of intimate partner violence in The Gambia - a cross sectional study of pregnant women

**DOI:** 10.1186/s12978-015-0023-x

**Published:** 2015-04-21

**Authors:** Patrick Idoko, Emmanuel Ogbe, Oley Jallow, Amaka Ocheke

**Affiliations:** Edward Francis Small Teaching Hospital, Banjul, The Gambia; Dalhatu Araf Specialist Hospital, Lafia, Nigeria; Jos University Teaching Hospital, Jos, Nigeria

**Keywords:** Intimate partner violence, Pregnancy, The Gambia

## Abstract

**Background:**

Intimate partner violence is an important public health problem that cuts across geographic and cultural barriers. Intimate partner violence refers to the range of sexually, psychologically and physically coercive acts used against women by current or former male intimate partners. The frequency and severity of violence varies greatly but the main goal is usually to control the victims through fear and intimidation. About 80% of Gambian women believe it is acceptable for a man to beat his wife thus encouraging the perpetuation of violence against women.

The objective was to ascertain the burden of intimate partner violence amongst pregnant women in Gambia.

**Methods:**

A cross sectional survey was carried out at Edward Francis Small Teaching Hospital, Banjul, The Gambia, on antenatal clinic attendees between October and December 2012, using a pre-tested structured interviewer administered questionnaire. All pregnant women were informed about the study at the antenatal booking clinic. Of the 161 pregnant women informed, 136 (84.5%) consented to take part and were recruited in the study. Descriptive analysis was done using the Epi info statistical software. Any pregnant woman booking for the first time during the period of the study was eligible to be recruited into the study.

**Results:**

Majority of enrolled participants (61.8%) reported intimate partner violence. Verbal forms of intimate partner violence were the commonest forms, with 12% requiring medical care on account of intimate partner violence and 3% prevented from seeking healthcare as a result of such violence.

**Conclusion:**

Intimate partner violence is common in The Gambia, West Africa and is a threat to women’s health.

## Background

Intimate partner violence (IPV) as defined by the World Health Organization (WHO) refers to the range of sexually, psychologically and physically coercive acts used against adult and adolescent women by current or former male intimate partners [1]. Even though men may also be victims of partner violence, the preponderance of evidence suggests that women are much more likely to be abused [1-3]. IPV can also occur in same-sex relationships and does not require sexual intimacy to occur. Traditionally, IPV is classified into 4 main types – sexual violence, physical violence, threats and psychological/emotional violence. However, these different types of IPV tend to occur together in a relationship. The frequency and severity of violence varies greatly but the main goal of the perpetrators is usually to control their victims through fear and intimidation.

IPV is an important public health problem that cuts across geographic and cultural barriers. Women who have experienced any type of IPV are more likely to have poor physical and mental health and use medical resources more [4]. Women’s reproductive and sexual health is also affected by such gender based violence. These problems include sexual dysfunction, pelvic inflammatory disease, sexually transmitted infections (including HIV) and infertility. These women are also at risk of unintended pregnancies and its sequelae – unsafe abortion, low birth weight babies, maternal and neonatal deaths. A systematic review of IPV and birth outcomes showed that adverse pregnancy outcomes are significantly more likely in women with a history of IPV [5]. These adverse effects include preterm delivery, chorioamnionitis, increased operative delivery, placental abruption and intrauterine foetal death [5-8]. In a review on human immunodeficiency virus (HIV) and domestic violence, it was reported that violence against female partners increases when a female partner is known to be HIV positive [9]. Potential ways in which HIV infection may be linked to intimate partner violence include: physical vaginal trauma from forced sex; limited capability to negotiate safer sex due to partner violence or threat of it; violence following disclosure of a positive HIV result and perpetrators more likely to engage in risky sexual behaviour [10].

Cultural norms in most traditional African societies do not support physical violence against women during pregnancy; however, anecdotal evidence seems to suggest that it is quite common. In a survey, IPV during pregnancy ranged from about 2% in Australia and Denmark to 13.5% in Uganda among ever pregnant, ever-partnered women [11]. The prevalence appears to be higher in Latin American and African countries compared to European and Asian countries [11]. In countries where studies have been done, it is generally believed that IPV is under estimated because it is under reported and there is a lack of standardized definition and tools for diagnosis.

Even though wife beating is a criminal offence in The Gambia, its occurrence is believed to be quite common. The police typically consider such incidents as domestic problems that can be settled by the families concerned [12]. More than 80% of Gambian women believe that a man is justified to beat his wife [13]. The Gambian laws prohibit rape and assault; however, spousal rape is not recognized by the law [12]. Anecdotal evidence suggests that violence against women during pregnancy by an intimate partner is quite common in The Gambia but there are no studies to ascertain the burden of the problem. Thus, it has not been recognized as a public health priority and is usually not treated as a fundamental human rights problem. Public health priorities in the country are weighted in favour of prevention of communicable diseases as well as maternal and child health strategies. However, the relationship between intimate partner violence and maternal and infant health has not yet been fully appreciated or studied in The Gambia.

Recognizing the burden of IPV, the American College of Obstetricians and Gynecologists opined that healthcare providers should be involved in screening for IPV, offer support and review options for prevention and referrals [14]. The antenatal clinic provides a setting for such screening, as this is most often, the only contact women have with the healthcare system in many developing countries. In spite of anecdotal evidence suggesting that IPV is very common in The Gambia, such screening is not available in the public health system.

IPV occurs more commonly in women in the reproductive age group [15]. Alcohol and substance abuse is thought to play a role in IPV [15]. Victims of IPV may resort to alcohol or substance abuse in attempt to help themselves. Perpetrators are more likely to cause serious injuries after taking alcohol [15].

This study aims to ascertain the burden of IPV amongst pregnant women and the factors associated with IPV in The Gambia. This will serve as a basis to advocate for larger studies and ultimately direct policy towards including antenatal screening for IPV as part of routine antenatal care.

## Method

The study was carried out at the Royal Victoria Teaching Hospital (RVTH), (now called Edward Francis Small Teaching Hospital) Banjul, The Gambia. RVTH is the only tertiary health centre in The Gambia serving its 1.7 million population. The country is a narrow strip of land bordered on 3 sides by Senegal and the Atlantic Ocean on the 4^th^ side. RVTH offers both primary health care services to women in the greater Banjul area as well as serving as the main referral centre for specialized tertiary care in maternity and reproductive health services.

A cross sectional survey was carried out on women who attended the antenatal clinic between October and December 2012. All pregnant women attending the antenatal clinic for the first time during the study period were eligible to be enrolled in the study. They were given information regarding the study by the clinicians at their first clinic visit. A structured interviewer administered questionnaire was used to collect data from all the pregnant women who consented to be a part of the study. Midwives in the antenatal clinic administered the questionnaire. The questionnaire was verbally translated into the local languages for those who did not understand English. Data was entered and analysed using Epi Info version 3.5.4 (CDC 2012). The questionnaire was pre-tested. Ethical approval for the study was obtained from the RVTH ethics board. Women who were found to be in severely abusive relationships were counseled and referred to the directorate of social welfare for further help.

## Results

One hundred and sixty one pregnant women who booked for antenatal care during the study period were given information regarding our study out of which 136 (84.5%) consented to be part of the study. Our sample consisted of the 136 women who consented to take part in the study. Table 1 shows the descriptive statistics of the study population and their partners. The minimum age of study participants was 14 years and the maximum was 51 years. The mean age was 26.6 ± 6.5 years. Our data show that 61.8% of pregnant women in the antenatal clinic had experienced IPV.

**Table 1:** **Descriptive characteristic of the participants and their partners**

**Variable**	**IPV**	**No IPV**	**P-value**
Mean age ± SD(years)	30.5 (6.2)	27.8 (6.8)	0.0596
Mean Parity ± SD	2.64 (2.07)	1.72 (1.86)	0.0417
Marital status	73	44	-
Married	1	0	
Divorced	10	8	
Single			
Educational level of	44	28	0.6631
woman	24	7	
None	13	16	
Basic	3	1	
High			
Tertiary			
Ethnicity	14	6	0.9456
Wolof	22	6	
Mandinka	18	7	
Fula	7	2	
Jola	23	31	
Others			
Religion	5	3	0.1127
Christian	79	49	
Muslim			
Partner’s educational	38	22	0.8151
level	14	6	
None	23	18	
Basic	9	6	
High			
Tertiary			
Occupation of Woman	31	7	-
Housewife	15	5	
(unemployed)	3	0	
Trader	9	8	
Farmer	26	32	
Civil servant			
Others			
Occupation of Partner	6	3	-
Farmer	21	5	
Busines	15	13	
Civil Servant	13	5	
Artisan	3	0	
None	25	26	
Others			

**Figure 1:**
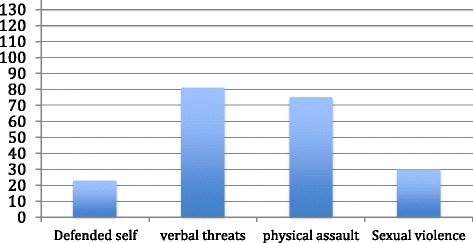
Experience of IPV by Gambian Women.

The most common forms of abusive behaviours experienced by the women were verbal (60%). While 55% reported physical violence, 22% of them had been forced to engage in a sexual act against their will (see Figure 1).

**Figure 2:**
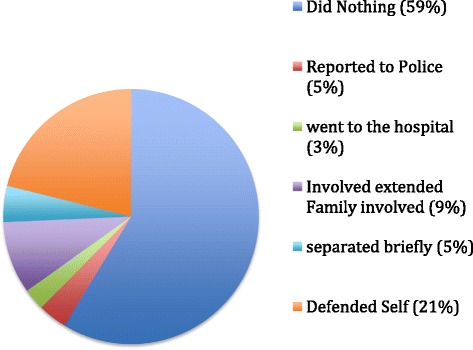
Women’s response to partner’s abusive behaviour.

Most of the women (59%) did nothing about the abusive behavior and only 4% reported the matter to the police (see Figure 2).

**Figure 3:**
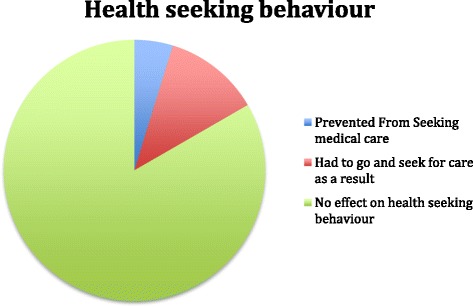
Health seeking behaviour of women as a result of experience of IPV.

In 5% of the victims, the perpetrators had prevented them from seeking health care while another 12% had to seek medical care as a result of the violence they experienced from their partner (see Figure 3).

Alcohol use by spouse was not associated with IPV but men who smoke cigarettes were 3 times more likely to perpetrate IPV (p-value 0.0038) (Table 2).

**Table 2:** **Relationship between substance use by partner and IPV**

**Substance**	**IPV**	**No IPV**	**OR**	**95% confidence interval**	**P-value**
Alcohol	9	4	1.44	0.42-4.93	0.56
Yes	75	48			
No					
Cigarette	42	13	2.98	1.40-6.34	0.0038
Yes	42	39			
No					

In 18 (20%) of the study participants who had experienced some form of violence, the violence was said to have decreased in the current pregnancy; it had increased in 10 (11%) and was unchanged in 56 (69%).

## Discussion

Our study showed that a majority of pregnant women (61.8%) had experienced IPV and being currently pregnant did not protect most of them (69%) from the abusive behaviour. While the most common form of abuse was verbal, more than half of the participants had experienced physical violence as well. The burden of IPV in this study is much higher than 7–20% reported in other studies [16-18]. However, Most of these other studies were focused on physical violence alone while our study looked at all forms of IPV. When physical violence alone is considered, the figure is still quite high. Reported frequencies of IPV tend to also vary depending on the methodology and definition of IPV used in the study. As the main referral hospital in the country, antenatal care patients are mostly high-risk pregnant women. This may have biased the result as IPV may be a contributing trigger in high-risk pregnancy. Furthermore, the sensitive nature of the subject matter makes underreporting a possible consideration in previous reports. The proportion of the various forms of violence experienced by our study subjects is similar to findings from other studies [19-21].

Being currently pregnant was not protective against IPV in our study. This is in keeping with findings from studies from other parts of the world that suggests that domestic violence often does not reduce in pregnancy [17,18]. Some studies from Africa seem to suggest that pregnant women are at a higher risk of experiencing gender-based violence because they are more likely to be in relationships compared to non-pregnant populations [22]. Indeed, most of the subjects in this study were married. Other studies from the West African sub-region seem to suggest that violence decreases during pregnancy by at least 10% [23]. Pregnancy does not prevent the occurrence of IPV however, there is conflicting evidence as to whether IPV decreases or increases during pregnancy [24]. A World Health Organization multicenter study showed that most women who reported physical abuse in pregnancy also reported being beaten prior to pregnancy although about half of the subjects in some sites reported that they were beaten for the first time in the index pregnancy [25].

While other African studies had shown that low level of education and being unemployed are risk factors for experiencing abuse [26,27], we did not find such an association. This is probably due to the fact that most of the subjects in our study had low educational levels and were unemployed. In the IMAGE study from South Africa, women who were economically empowered through different means like credit extension managing loans reported reduced risk of IPV [21].

This study also showed that alcohol use by the spouse is not significantly associated with partner violence, which is in contrast to studies that have shown otherwise [26-28]. This may be explained by the fact that drinking alcohol is a culturally unacceptable practice in The Gambia leading to very few respondents admitting that their partners drink alcohol. An association between IPV and cigarette smoking has been reported by other studies like ours [29,30].

Most of the subjects in our study did not do anything about the abusive bahaviour. This practice tends to perpetuate the practice of gender-based violence, as the perpetrators often do not get reprimanded for their actions. Our study makes an argument for including screening for IPV in routine antenatal care in The Gambia. However, larger population based studies may be necessary to validate our findings.

The main limitation of this study is that it is a hospital-based study whose findings should be interpreted with caution when applied to the general Gambian population. The study centre is the only tertiary health facility in the country and the main referral hospital in the country. Thus, our study population is composed of a higher proportion of high-risk pregnant women, which may have biased our findings. However, this opens up the need for further research on IPV in the country. There is also the need for studies looking at the health consequences of IPV in The Gambia.

## Conclusion

Intimate partner violence is very common in The Gambia. Most women accept violence as a way of life and do nothing about it. The culture of silence on this very practice needs to be broken to encourage more discussion and intervention on this matter.

## Abbreviations

CDC: Center for Disease and Control
HIV: Human immunodeficiency virus
IPV: Intimate partner violence
RVTH: Royal Victoria Teaching Hospital
WHO: World Health Organization
